# The Role of Depression and Anxiety in Frail Patients with Heart Failure

**DOI:** 10.3390/diseases7020045

**Published:** 2019-06-19

**Authors:** Elisabeta Ioana Hiriscau, Constantin Bodolea

**Affiliations:** 1 Department of Nursing, Medical Faculty, University of Medicine and Pharmacy “Iuliu Hatieganu”, 400083 Cluj-Napoca, Romania; 2 Department of Anesthesiology and Intensive Care, Medical Faculty, University of Medicine and Pharmacy “Iuliu Hatieganu”, 400006 Cluj-Napoca, Romania; cbodolea@gmail.com; 3 ICU Department, Clinical Municipal Hospital, 400139 Cluj-Napoca, Romania

**Keywords:** anxiety, depression, frailty, heart failure, psychological factors

## Abstract

An increased interest regarding the impact of frailty on the prognosis of cardiovascular disease (CVD) has been observed in the last decade. Frailty is a syndrome characterized by a reduced biological reserve that increases the vulnerability of an individual in relation to stressors. Among the patients with CVD, a higher incidence of frailty has been reported in those with heart failure (HF). Regardless of its conceptualizations, frailty is generally associated with negative outcomes in HF and an increased risk of mortality. Psychological factors, such as depression and anxiety, increase the risk of negative outcomes on the cardiac function and mortality. Depression and anxiety are found to be common factors impacting the heart disease and quality of life (QoL) in patients with HF. Depression is considered an independent risk factor of cardiac-related incidents and death, and a strong predictor of rehospitalization. Anxiety seems to be an adequate predictor only in conjunction with depression. The relationship between psychological factors (depression and anxiety) and frailty in HF has hardly been documented. The aim of this paper is to review the reported data from relevant studies regarding the impact of depression and anxiety, and their effects on clinical outcomes and prognosis in frail patients with HF.

## 1. Introduction

The importance of investigating cardiovascular disease (CVD) in frail patients has been identified as a priority for care, the main interest being the recognition and implications of frailty on the cardiac outcomes [1]. Frailty is defined as a syndrome having as its main characteristics the reduced biological reserve and functional decline of the whole organism, which lead to an increased vulnerability of the individual, even to minor stressors [2]. Although frailty is related to chronological age and is often referred to older people, not all frail people are aged. Some medical conditions could increase the frail status, even in younger population.

Identifying frail syndrome in CVD patients or in those undergoing cardiac surgery has relevant implications for clinical practice. Clinical outcomes of CVD tend to be worse among frail individuals who have a higher rate of morbidity and mortality [3]. The presence of frailty may reduce the benefits of surgery in some cardiac diseases because of competing risks. Falls, functional impairment, health resource utilization, and hospitalizations are conditions related to a pre-frail and frail status, and to adverse outcomes in CVD. Therefore, a frailty assessment should be included in the global evaluation of CVD patients in order to improve the clinical decision-making and planning of therapeutic actions.

A broad range of psychological and social characteristics have been investigated in relation to CVD and risk-related factors. Depression, anxiety and/or psychological distress have also been related to a higher risk of coronary disease, sudden cardiac death, stroke, and all-cause mortality [4].

The negative impact of depression among cardiac patients has been long clinically recognized and reported in many studies [5]. Major depressive disorder, current depressive symptoms, and a history of depression are all associated with an increased risk of CVD morbidity and mortality. Depression in patients with CVD is associated with a worse health-related quality of life (QoL). QoL, recurrent cardiac events, a risk of hospital readmissions, and a risk of functional decline and mortality, with a long-term impact on the cardiac function outcomes. High rates of sudden cardiovascular death in depressed patients have been also reported [6,7]. Among coronary patients, higher levels of anxiety have been associated with a poorer prognosis and greater recurrence of cardiac events post-myocardial infarction [8,9].

The relationship between frailty, psychological factors and their cumulative impact on the outcomes and prognosis in CVD has been paid increasing attention in the last decade. Still, little literature is available in this regard. The investigation of how frailty, depression and anxiety influence the outcomes and prognosis of cardiac disease may contribute to a broader understanding and an increasing awareness on the part of clinicians and healthcare professionals to include an assessment of the psychological factors in the global cardiac evaluation.

## 2. Background and Definition of Key Concept

### 2.1. Frailty and Heart Failure

Heart failure has become a major public health problem and the leading cause of morbidity, hospitalization and mortality in older people. Frailty was associated with clinical cardiovascular disease, most strongly with HF [10,11]. Eighty percent of patients with HF are elderly (65 years and older), are more likely to be women, and are more likely to have frailty, a higher burden of comorbidities, and a preserved left ventricular ejection fraction (LVEF) [12]. Vitale et al. reported that up to 79% of patients with HF are frail, independent of their age or New York Heart Association (NYHA) functional classification [13]. Frailty is associated with a poor quality of life, a higher risk of disability, a need of readmission in the hospital, medical assistance, the utilization of healthcare resources, and mortality [14].

Frailty has been conceptualized in two ways—a phenotype model and an accumulated deficit model [15,16].

The first model is proposed by Fried and is known as the frailty phenotype. According to this view, frailty is defined as a distinct clinical syndrome based on physical functioning and on the biological/physiological state. According to this model, a frail individual presents three or more of the following five physical components: weakness, poor endurance and energy (self-reported exhaustion), slowness, low physical activity level, and unintentional weight loss. Several studies have shown that the prevalence of HF increased from 1.8% in the non-frail to 14.0% in the frail group [12]. In elderly patients with HF who have frailty, the rate of 1-year mortality is about 16.9% compared to those who do not (4.8%); respectively speaking, the rate of hospitalization is higher compared with non-frail individuals (20.5% vs. 13.3%) [17]. Frailty is found in HF patients of all ages and is strongly related to a poor response to treatment, worse functional outcomes, and an increased mortality [18].

Neurohormonal, metabolic, immunologic and musculoskeletal disorders may lead to an increased catabolic state that is typical of the frail phenotype in HF. As result of this process, frail patients will lose muscular mass, becoming sarcopenics; the increased oxidative stress and cytokine activation will predispose them to cardiac cachexia, with a consequent weakness, fatigue and reduced resistance to stressors and to survival. Cardiac cachexia represents the terminal phase of body wasting seen in advanced stages of HF, and along with body wasting it is associated with a higher mortality rate [19].

The second model defines frailty as a multidimensional construct based on physical, psychological, and social components [20]. The underlying idea of this approach is to consider, in the conceptualization of frailty, not only the physical problems but also the psychological and social aspects that together define the global functioning of an individual [21,22]. In accordance with this model, the definition of frailty as a ‘’dynamic state affecting an individual who experiences losses in one or more domains of human functioning (physical, psychological, social), which is caused by the influence of a range of variables and which increase the risk of adverse outcome”, is currently supported by the researchers’ community [16]. This view is also in line with the assumptions of the bio-psycho-social model that supports the idea that a proper approach and treatment of the disease should also involve the psycho-social aspects that, along with physical health, determine the individual’s QoL [23].

According to this model, a progressive series of damages across multiple organ systems are identified, ranging from functional decline to disability and death. The deficits relate mainly to mobility, strength, balance, motor processing, cognition, nutrition, physical activity, and they affect the capacity of the individual to cope with distress in a major way [24]. Associated to this condition, depressive symptoms and anxiety may negatively affect the health outcomes, onset of disability and reduced QoL. Furthermore, isolation, loneliness, the loss of social contacts or the absence of social support are related to negative disease outcomes and mortality [25].

### 2.2. Assessment of Frailty

In order to identify frail individuals, rapid and simple screening tests have been developed and validated, differing mainly in what they are supposed to measure in accordance with the nature of two contrasting conceptual models: the frailty phenotype, or physical frailty, and the frailty index, or deficit accumulation. The most commonly used, depending on the frailty conceptualization, are the FRAIL Questionnaire screening tool, the Cardiovascular Health Study Frailty Screening Scale (Fried criteria), the Clinical Frailty Scale (CFS), frailty indices, and the Edmonton Frail Scale.

The FRAIL Questionnaire screening tool takes into consideration five domains that are equally weighted in the frailty assessment: fatigue, resistance, ambulation (slow walking speed), illnesses, and loss of weight (5% or more in the past year). Individuals with two deficits are considered pre-frail, and those with three or more deficits are classified as frail [26].

The Fried criteria for frailty assess physical characteristics or phenotypes, which include five domains: unintentional weight loss (4.5 kg or more in the last year), exhaustion (self-reported), a low physical activity, weakness (low grip strength), and the walking speed. The classification of frail individuals is similar to that of the FRAIL Questionnaire [15].

The CFS is a global clinical assessment of frailty based on the physical function and level of independence with activities of daily living (ADL). Each point on the scale corresponds to a visual chart and a written description of frailty. Scoring is based on clinical judgment and ranges from 1 (very fit) to 9 (terminally ill) [16].

Frailty indices are based on the deficit model to measure frailty and are useful for estimating the related risk for adverse health outcomes [27].

The Edmonton Frail Scale (EFS) assesses the following dimensions: cognition, general health status, self-reported health, functional independence, social support, nutrition, mood, continence, and functional performance. Depending on the scores obtained out of maximum of 17 points, the frail individuals are classified as not frail (0–5), apparently vulnerable (6–7), mildly frail (8–9), moderately frail (10–11) and severely frail (12–17) [28].

### 2.3. Psychological Factors and Heart Failure

#### 2.3.1. Depression

Depression is considered an independent risk factor for cardiac morbidity and mortality. Among the elderly, it is closely related to cognitive decline and dementia. Moreover, depression is a strong predictor of readmission in hospital for patients with congestive heart failure (CHF), independent of the initial severity of illness [29,30]. In the treatment of elderly patients with HF who have a high level of dependency in daily activities, it is important in the assessment to consider the presence of factors associated with a poor prognosis, such as comorbidities and geriatric syndromes, depression, and cognitive impairment.

The prevalence rate of depression in patients with HF is 36% for increased depressive symptoms and 20% for major depressive disorder (MDD). Among patients admitted in the hospital with a HF exacerbation and diagnosed with MDD, less than half had presented a remission of symptoms. Depression is also linked with higher rates of readmission in the hospital, especially, in younger patients with CHF and in the elderly; this might be due to their refusal to comply with medical treatment, resulting in the worsening of their condition, which may require hospitalization [31,32,33].

Depression and anxiety often coexist and may be a common factor impacting heart disease. The effect of combined depression and anxiety is reflected upon by an increased risk of hospital readmission or death compared to patients without any symptoms. Coexisting depression and anxiety (but not depression or anxiety alone) was an independent predictor of a cardiac event [34,35].

#### 2.3.2. Anxiety

Anxiety is referred to as a condition that frequently appears along with depression [36]. Although there is a little data in the literature investigating the impact of anxiety in patients with HF, anxiety disorders and cardiac health are strongly related. Generalized anxiety disorder (GAD), panic disorder, and post-traumatic stress disorder (PTSD) have been associated with an increased risk of coronary artery disease (CAD) or HF [37,38]. Compared to depression, elevated rates of anxiety have been reported in over 50% of patients with HF, and 13% of cases met the criteria for an anxiety disorder. However, this evidence was supported only by limited research that explored the prevalence, nature and impact of anxiety in patients with HF. Some research data showed that the prevalence estimates of anxiety ranged from 6.3% to 72.3% in HF patients, compared to other cardiovascular pathologies. A lower prevalence estimate of anxiety was found in studies measuring specific anxiety disorders, and the upper range was found in patients following an invasive procedure, as well as in patients with HF compared with other cardiovascular conditions. Regarding the gender differences, higher rates of anxiety were reported in females’ groups with HF than in males’ after acute cardiac events [39].

### 2.4. Frailty and Psychological Factors in Heart Failure

There are very limited data in the literature about the relationship between the frail status, and depression and anxiety in patients with HF. Research in this area emphasized the idea of the early detection of the frail status associated with any mood changes, anxiety or other psychosocial correlates in order to prevent a functional decline and in order to improve the quality of life (QoL) in patients with HF. Research that investigated the association between the frail status and cardiac disease showed a prevalence ranging from 15% to 74% in patients with HF [40,41]. The studies investigating the role of psychosocial support in hospitalized frail HF individuals highlighted that these patients have an increased likelihood of incurring negative outcomes (length of hospital stay, re-hospitalization, and mortality) in the absence of a supportive environment, compared to frail people with good psychosocial functioning [34].

In this paper, we examine the studies that explore the impact of psychological factors–depression and anxiety–in frail patients with HF on clinical outcomes (readmission and mortality). We will present and discuss the findings of the studies that were identified as relevant for the proposed topic and then formulate some conclusions.

## 3. Materials and Methods

This review was reported according to the preferred reporting items for systematic reviews (PRISMA) guidelines. The analysis is based on previous published studies; thus, no ethical approval and patient consent are required.

### 3.1. Search Strategy

**Figure 1:**
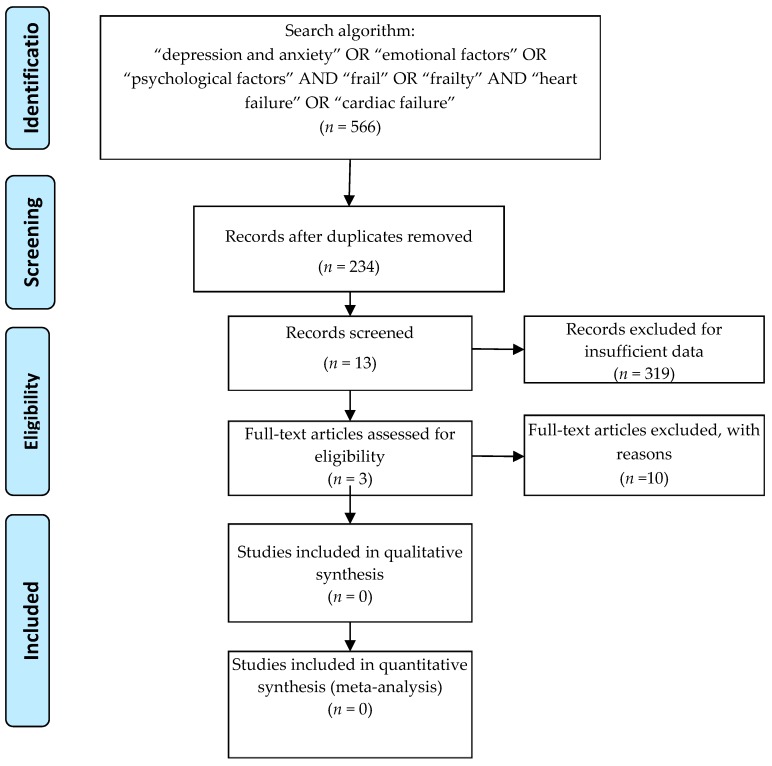
Selection of the publications.

Potentially relevant studies were identified from electronic databases, including Medline (1966 to April 2019), PubMed (1966 to April 2019), Embase (1989 to April 2019), ScienceDirect (1985 to April 2019), and Ovid (2007 to April 2019). Searches included literature found from the database’s origin to April, 2019. The following keywords were used in combination with Boolean operators AND or OR: “depression and anxiety” OR “emotional factors” OR “psychological factors” AND “frail” OR “frailty” AND “heart failure” OR “cardiac failure”. We used the search driver only in English. The search process was performed as presented in Figure 1.

### 3.2. Inclusion and Exclusion Criteria

The selection of the studies was based on the following criteria:

- Participants: only published articles enrolling adult patients (diagnosed) with HF, hospitalized or in an ambulatory setting.
- Assessment: HF participants screened for frailty and for depression and anxiety, regardless of the method used.
- Comparisons: control group of non-frail HF patients.
- Outcomes: clinical outcomes (readmission and mortality), functional status, QoL.
- Study design: observational studies, cross-sectional studies, randomized control studies.

### 3.3. Selection Criteria

One author screened the literature search for depression and anxiety, and for frailty in HF. Each author independently reviewed all of the abstracts of the resulting citations and checked them to identify relevant articles, according to the above-mentioned search algorithm. Every full text of the studies that met the inclusion criteria was screened by the authors. Disagreements regarding the eligibility of articles were resolved by consensus building after reading the full text.

### 3.4. Data Extraction

One author extracted data from the included studies. The following data was extracted and recorded in a spreadsheet: first author names, publication year, sample size, characteristics of the sample, and assessment methods of frailty, depression and anxiety (see Table 1). The primary outcomes were the readmission rate and mortality. The secondary outcomes were the functional decline and QoL.

### 3.5. Quality Assessment of the Articles

The quality assessment of the included studies was performed by one author (see Table 2) who used the Quality Assessment Tool for Observational Cohort and Cross-Sectional Studies, NHLBI (National Heart, Lung and Blood Institute), NIH (National Institute of Health).

## 4. Results

### Search Result—Study Characteristics

After removing duplicates (234), 319 records were screened to identify the studies on HF which included an assessment for frailty and depression and anxiety. 306 records were excluded from the analysis and were organized under the following categories:

- Studies on HF only = 14
- Studies on HF and frailty = 7
- Studies on frail individuals only = 31
- Recommendations/Guidelines/Position Statement = 17
- Review articles on different topics = 9
- Studies on elderly/geriatrics individuals = 53
- Studies on QoL (on different categories of individuals) = 16
- Pharmacological studies = 12
- Studies other than HF = 63
- Studies other than diseases = 49
- Studies on physical activity/rehabilitation programs = 22
- Frailty and Depression, but not anxiety = 8
- Anxiety and Depression, but not frailty = 5

Three publications were finally included in the present review. The types of publications included in this review are in accordance with the scope of the present article. We included one prospective observational study, one prospective cross-sectional study and one observational cohort study in the analysis. The full-text articles (10) excluded from the analysis did not satisfy the inclusion criteria.

## 5. Discussion

The purpose of this paper was to highlight the impact of depression and anxiety, and of frailty on the clinical outcomes (readmission and mortality, functional decline, QoL) in HF patients. Due to the limited data published on this subject, the mission of the article was only partially accomplished. In this paper we covered, under the global term psychological distress, the level of depression and anxiety perceived by an individual.

Generally, frailty is considered highly prevalent in elderly patients with CVD, being mainly related to a high risk of adverse outcomes, including disability, lower quality of life, hospitalization, admission in nursing facilities, and mortality [18]. The prevalence of frailty in HF patients is higher than that in the general population and ranges from 15% to 74%. Knowing that frailty can predict negative outcomes in HF patients that can result in disability and/or mortality is of great importance in clinical practice to identify the individuals at risk of frailty and to implement the appropriate measures to reverse this condition’s effects, if possible. The frail HF patients experience different levels of psychological distress due to, on the one hand, the prognostic, and on the other hand the potential complications that could occur in the course of the disease.

The prevalence of depression is high and may be increasing in individuals with HF. Evidence shows a prevalence of depression among patients with HF that ranges from 15% to 36% [42]. Moderately and severely depressed patients with HF are reported to have a significantly higher mortality than HF patients with mild depression or non-depressed patients. Those reporting severe depression are four times more likely to die within 2 years compared with non-depressed patients. Higher depression rates have been identified in the hospitalized HF patients (ranging from 13.9% to 77.5%) compared to outpatients with HF (ranging from 13% to 48%). Three predictors for depression in CHF hospitalized patients have been identified: functional impairment, the severity of illness and comorbid psychiatric disorder [43]. Hospitalized depressed patients with CHF, untreated for a depressive condition, result in a worse prognosis, higher demands on health service use and a higher readmission rate associated with increased adverse clinical outcomes. Depression is also related to the severity of HF symptoms; the baseline functional status, including a limitation in the activities of daily living and dyspnea at rest, are reported as being strongly related to depression [44]. Friedman and Griffin reported significant correlations between depression severity and increased physical symptoms or decreased physical functioning [45]. In the study conducted by Uchmanowicz and Gobbens, the frail syndrome has been correlated with a higher score of depression and anxiety [46]. The average values of the HADS-anxiety and HADS-depression were significantly higher in the frail group compared to the non-frail group of patients. Significant positive correlations have been found between the TFI scores and HADS-anxiety (r = 0.60, *p* < 0.001), and the HADS-depression (r = 0.66, *p* < 0.001) results. This means that increased levels of frailty measured by the TFI scale are accompanied by an increase in the level of anxiety and depression. The results from this study showed a deterioration of QoL in HF patients who had an increase in their level of anxiety and depression, as well as a high frailty-related score. Depression is also related with a higher frequency of readmissions in the hospital in HF patients versus those without depressive symptoms. No data regarding the impact of combined frailty and coexisting depression and anxiety on the clinical outcomes in HF patients, such as the readmission rate or mortality, have been provided in this study.

Depression and anxiety are strongly related to hospitalization, the use of health care resources and recurrent events involving frequent readmissions [41]. The prevalence of depression and anxiety is high in chronic patients with HF (10–60% depression; 11–45% anxiety). Comorbid depression and anxiety are associated with an increased mortality and health care utilization.

Anxiety appears to be a less investigated dimension in the study of HF. The existing evidence suggests a prevalence of anxiety as high as 63% in HF patients. Few researchers have reported that the presence of anxiety symptoms is an independent predictor of a worsening functional status and more frequent hospitalizations. The association of anxiety with HF patient outcomes has been little investigated, and the results are inconsistent. It appears that anxiety predicted a functional status at 1 year in patients with HF, but not rehospitalization or mortality [47]. Other research findings showed that in patients with recent acute myocardial infarction and depressed left ventricular function, anxiety was associated with a higher incidence of adverse cardiac events and cardiac death in the subsequent 6–10 years [41,46]. High levels of depression and anxiety are considered risk factors for first and subsequent events (readmissions in the hospitals) in HF patients, as presented in the study conducted by Sokorelli et al. [48]. It has been shown that the first adverse event in HF patients is related to the presence of both medical and psychological factors. The results of the study showed a higher rate of unplanned readmissions due to at least one cardiac event (52%) and a mortality rate of 19% at a 1-year follow-up. The presence of frailty, anxiety and depression were powerful predictors of the outcome of both the first and recurrent events. Some research data showed a statistical relationship between the presence of frailty and the need for HF hospitalization, but not a statistically significant relationship between depressive symptoms and need for HF hospitalization.

A moderate-to-severe depression and anxiety, cognitive deterioration and the presence of frailty along with medical circumstances such as an increasing age, past history of a cardiac disease, LVEF < 40%, and increasing urea and creatinine at discharge, are all associated with the risk of a first event. The HF patients with a moderate-to-severe level of depression have a 1.7 times higher risk for the first event and a 1.8 times higher risk for the recurrent events, while those with a similar level of anxiety have, respectively, a 1.7 and 1.4 times higher risk [48]. In this study, it was found that both depression and anxiety are related to the risk of recurrent events [49]. These findings contradict other data reported that has not shown any association between anxiety and mortality, with only depression being associated with negative outcomes and mortality [44].

Denfeld et al. highlighted the importance of also assessing affective symptoms in HF patients, besides physical frailty [50]. The results showed that physically frail patients with HF have significantly worse dyspnea and wake disturbances as physical symptoms, and a high-related level of depression, compared with those who are not physically frail. Despite the evidence that high levels of depression in HF frail patients interfere with the physical status, having a negative potential upon the cardiac functioning, anxiety has not been identified in such a way in this study. The authors emphasized the important role of a physical frailty assessment in clinical practice, along with both physical and affective symptoms experienced by patients with HF. The study conducted by Son and Seo showed that depressive symptoms are the most critical predictor of physical frailty in older adults with HF [51]. The detection of depressive symptoms in older adults with HF may alarm clinicians about the risk of physical frailty in this population [52]. Therefore, depressive symptoms should be assessed and managed as a comorbid condition for monitoring physical frailty in older adults with HF.

Including both types of symptoms in the assessment of HF patients may raise awareness in the early identification of patients with more advanced HF, especially in circumstances where worse symptoms are associated with an impaired cognition and physical capacity. A worst prognostic is given by the association of frailty with comorbidities that could lead to physical deterioration, cognitive impairment and a psychological dependence resulting in a high risk of non-compliance with HF treatment. Any depressive condition presented in HF patients alters their perception of QoL and affects the health status which, in turn, may negatively impact the patient’s self-care. Zhang et al. showed that psychological distress, including stress, anxiety and depression, accounted for 13% of the variability of the overall QoL total score (the second of seven clusters of importance) [53].

## 6. Conclusions

Psychological distress and the frailty status play a key role in predicting the clinical outcomes in patients with heart failure. Still, limited data are available in this regard, and further research in this field is required.

Frail syndrome in HF patients is an independent predictor for adverse events and for mortality. In HF patients, high levels of depression and anxiety, along with frailty, may impede the recovery and worsen the prognostic of the disease without an appropriate management of these comorbidities. As risk factors for the occurrence of the recurrent events, depression and anxiety events, along with frailty and cognitive impairment, negatively impact the QoL in HF patients. In the absence of carefully monitoring the emotional status related to any exacerbation of the disease, the long-term results of the therapeutic interventions might be less efficient in frail patients with HF, compromising their QoL and increasing the risk of rehospitalization and mortality. Psychological support should be focused on the limitation of the negative effects of depression and anxiety on the prognosis of the disease and of survival in patients with heart failure.

## Figures and Tables

**Table 1:** Publications included in the review.

Studies	Reference Type	Location	Sample	Mean Age	Characteristics of Population	Assessment	
						Frailty	Depression and Anxiety
Sokoreli 2018	Ongoing prospective observational study	UK	779 participants hospitalized for HF	75 (67–82)	treatment with loop diuretics and LVEF ≤ 40	1. questions about having trouble with bathing and dressing 2. TUG test < 10 s normal > 20 s abnormal	HADS questionnaire Scoring: < 7 no depression or anxiety 8–10 mild depression or anxiety > 11 moderate-to-severe depression or anxiety
Denfeld 2017	Prospective cross-sectional study	USA	49 participants (outpatient and inpatient) scheduled for a right heart catheterization	57.4 ± 9.7	NYHA III or IV, non-ischemic HF	1. Shrinking (self-report) 2. Weakness (5-repeat chair stands) 3. Slowness (4 m Gait speed) 4. Physical exhaustion (FACIT-F) 5. Level of physical activity (one question)	Depression: 9-item PHQ (valid and reliable measurement of depression in HF) Scoring (range 0–27): 10 = moderate depression > 10 greater depression Anxiety: 6-item BSI (valid and reliable measure of anxiety in HF) Scoring (range 0–4): 4 = worse anxiety
Uchmanowicz 2015	Single-centre observational cohort study	Poland	100 participants	non-frail, 62.3 ± 6.2 years; frail, 67.9 ± 10.7 years	> 60 years, with a diagnosis of HF, enrolled from clinic	TFI scale (Polish version) with 15-self-reported questions regarding: 1. physical domain (0–8 points) 2. psychological domain (0–4 points) 3. social domain (0–3 points)	HADS questionnaire Scoring: < 7 no depression or anxiety 8–10 mild depression or anxiety > 11 moderate-to-severe depression or anxiety

BSI, Brief Symptom Inventory; FACIT-F, Functional Assessment of Chronic Illness Therapy Fatigue Scale; HADS, Hospital Anxiety and Depression Scale; LVEF, Left ventricle ejection fraction; HF, heart failure; NT-proBNP, N-terminal pro-B-type natriuretic peptide; PHQ9, Patient Health Questionnaire; TFI, Tilburg Frailty Indicator; and TUG, ‘timed up and go’.

**Table 2:** Quality Assessment Tool for Observational Cohort and Cross-Sectional Studies, NHLBI (National Heart, Lung and Blood Institute), NIH (National Institute of Health).

Studies	Sokorelli, 2018			Denfeld, 2017			Uchmanowicz, 2015		
Criteria	Yes	No	Other (CD, NR, NA) *	Yes	No	Other (CD, NR, NA) *	Yes	No	Other (CD, NR, NA) *
1. Was the research question or objective in this paper clearly stated?	x			x			x		
2. Was the study population clearly specified and defined?	x					small sample			small sample, recruited from a single center, 89% of the patients were frail
3. Was the participation rate of eligible persons at least 50%?			NR			NR			NR
4. Were all the subjects selected or recruited from the same or similar populations (including the same time period)? Were inclusion and exclusion criteria for being in the study prespecified and applied uniformly to all participants?	x			x			x		
5. Was a sample size justification, power description, or variance and effect estimates provided?		x		x			x		
6. For the analyses in this paper, were the exposure(s) of interest measured prior to the outcome(s) being measured?	x			x			x		
7. Was the timeframe sufficient so that one could reasonably expect to see an association between exposure and outcome if it existed?	x			x			x		
8. For exposures that can vary in amount or level, did the study examine different levels of the exposure as related to the outcome (e.g., categories of exposure, or exposure measured as continuous variable)?	x			x			x		
9. Were the exposure measures (independent variables) clearly defined, valid, reliable, and implemented consistently across all study participants?	x			x			x		
10. Was the exposure(s) assessed more than once over time?			NA			NA			NA
11. Were the outcome measures (dependent variables) clearly defined, valid, reliable, and implemented consistently across all study participants?	x			x			x		
12. Were the outcome assessors blinded to the exposure status of participants?			NA			NA			NA
13. Was loss to follow-up after baseline 20% or less?		x				NA			NA
14. Were key potential confounding variables measured and adjusted statistically for their impact on the relationship between exposure(s) and outcome(s)?	x			x			x		

* CD, cannot determine; NA, not applicable; NR, not reported.

## Abbreviations

CVD	cardiovascular disease
HF	heart failure
LVEF	left ventricular ejection fraction
QoL	quality of life
CHF	congestive heart failure
MMD	major depressive disorder
GDA	generalized anxiety disorder
PTSD	post-traumatic stress disorder
CAD	coronary artery disease
PCS	physical component scale
MCS	mental component scale
